# Is there a role for telemedicine in disaster medicine?

**DOI:** 10.1186/s13054-014-0646-2

**Published:** 2014-11-27

**Authors:** Felipe Piza, Milton Steinman, Sergio Baldisserotto, Renata Albaladejo Morbeck, Eliezer Silva

**Affiliations:** Hospital Israelita Albert Einstein, Avenida Albert Einstein, 627/701, Morumbi, São Paulo 05651-901 Brazil; Hospital Universitario de Santa Maria, Av. Roraima, Prédio 22, Campus, Bairro: Camobi, Santa Maria, RS CEP: 97105-900 Brazil

Early on 27 January 2013, 242 people were killed in a nightclub fire in southern Brazil (Santa Maria) after a band’s pyrotechnic show set the establishment ablaze. The survivors were confined inside the nightclub for an unknown period of time. A few hours later, Hospital Israelita Albert Einstein, a level-one hospital in São Paulo (Brazil), located 1,247 km (775 miles) from Santa Maria, managed to organize a humanitarian aid and disaster relief mission.

A regional telemedicine (TM) hub was installed in the Hospital Universitario de Santa Maria, which is one of the main sites that received injured victims. The miniaturized mobile TM platform TES (Transportable Examination Station; GlobalMed Inc., Scottsdale, AZ, USA) was chosen as a practical and lightweight device (Figures 1 and 2). This device operated uninterrupted for 2 weeks after the disaster with at least two videoconferences a day (Figure 3).

**Figure 1:**
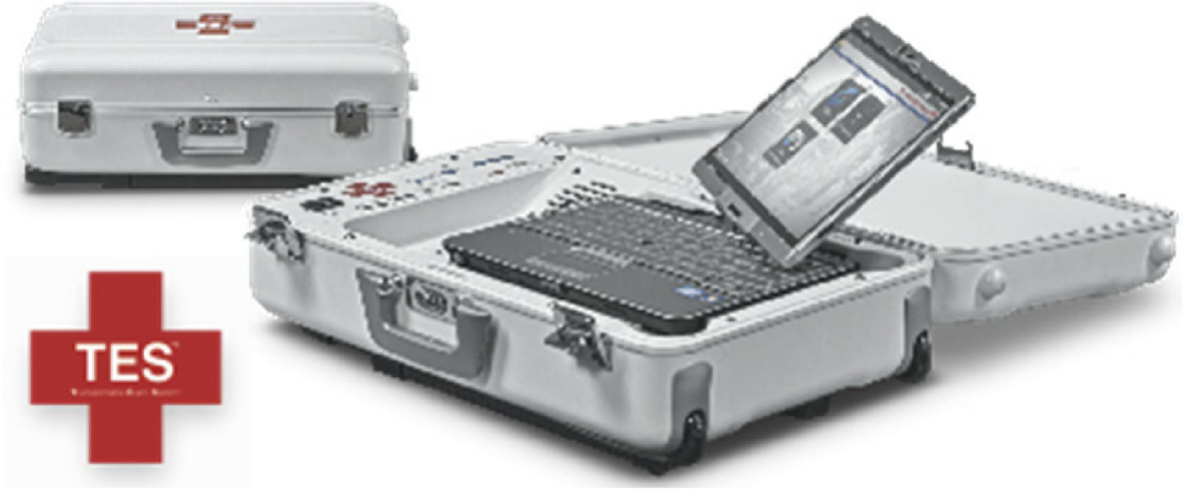
**Miniaturized mobile telemedicine platform TES® (Transportable Examination Station; GlobalMed® Inc., Scottsdale, AZ, USA).**

**Figure 2:**
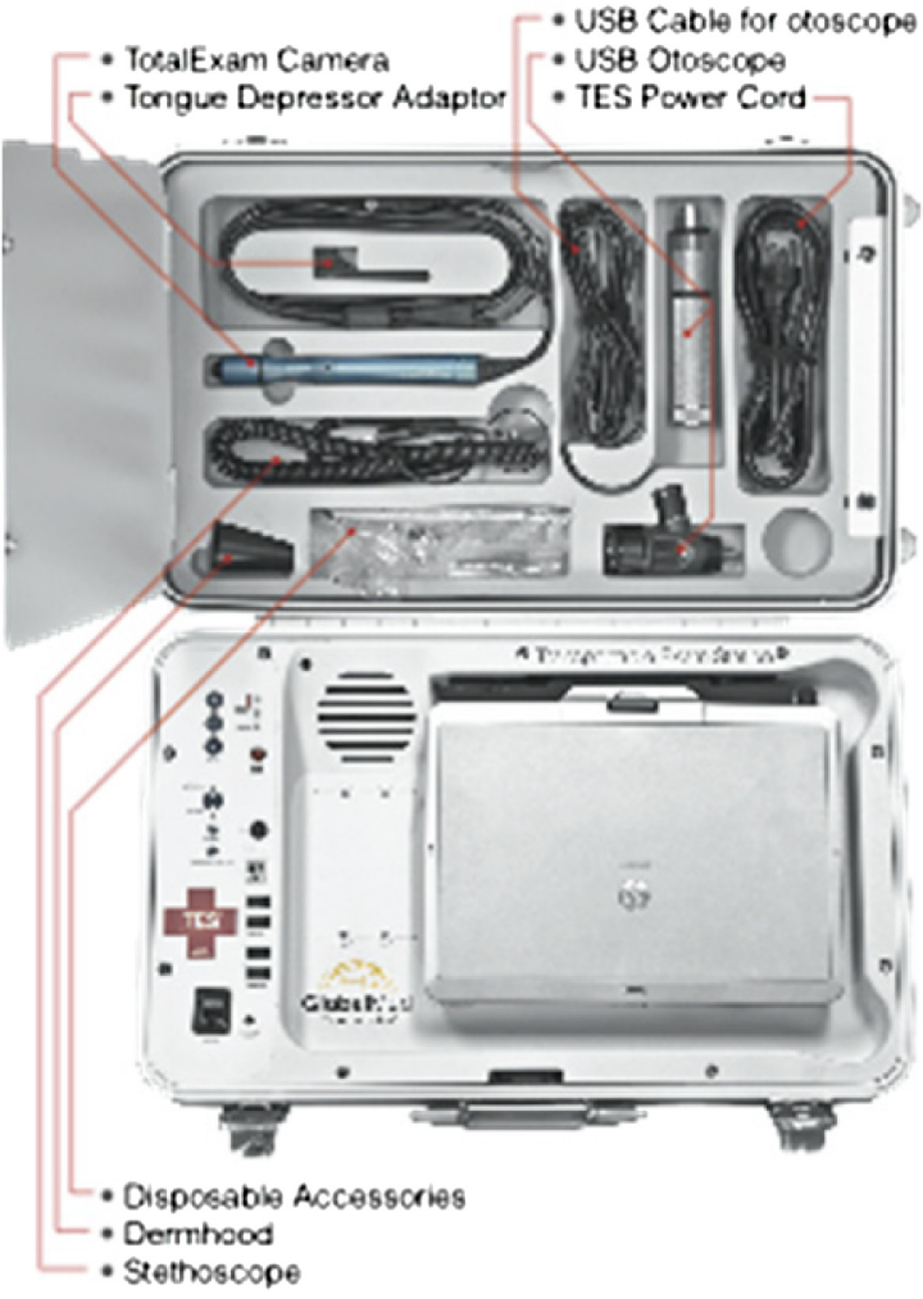
**Miniaturized mobile telemedicine platform TES® (Transportable Examination Station; GlobalMed® Inc., Scottsdale, AZ, USA).** USB, Universal Serial Bus.

**Figure 3:**
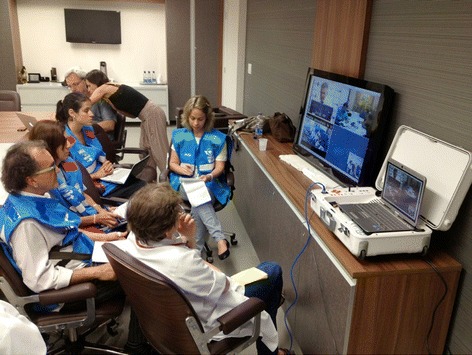
**Health-care providers during videoconferences.** Telemedicine inside the Hospital Universitario de Santa Maria the following day after the disaster with people from the Health Ministry, local physicians and staff discussing, through telemedicine, with specialists about patients who were in intensive care after the disaster and in need of supplies.

Unfortunately, 242 people died at the scene. Approximately 620 people needed immediate medical attention and were distributed among nearby hospitals. Approximately 80 victims required admission because of respiratory failure, burn injuries, carbon monoxide and cyanide poisoning, and minor trauma. Mechanical ventilation was performed on 42 patients.

TM allowed emergency staff to discuss these challenging cases with specialists from around the world. Ventilation strategies and bronchoscopy interventions in injured airways were some of the primary issues. Daily ‘toilet bronchoscopy’ was performed during the first 2 days (as suggested during the conferences) to improve the release of smoke with marked improvement.

The ceiling foam of the nightclub was composed of polyurethane, which during the fire released cyanide and carbon monoxide that accounted for the majority of the deaths. TM helped to diagnose and manage these patients, and many other supplies were sent after TM sessions.

None of the 80 admitted patients died in Santa Maria hospitals. TM encompasses the diagnosis, treatment, monitoring, and education of patients and provides convenient, site-independent access to expert advice and patient information [1-3].

The limitations of TM should be considered, especially when natural disasters severely damage the local infrastructure and networks, thus threatening internet connections. Although TM can provide consultation and decision making, it cannot help in procedural skills [4].

We consider that there is a role for TM in disaster medicine. Mobile health addresses complex issues at the intersection of medicine, public health, and logistics and improves access to external specialists.

## Abbreviation

TM: Telemedicine
